# Suicide in sculpture: qualitative thematic analysis and psychiatric perspective

**DOI:** 10.1192/bjo.2026.11029

**Published:** 2026-04-24

**Authors:** Hanife Neris Yüksel, Deniz Doruk Çamsarı, Taner Çamsarı, Ulaş Mehmet Çamsarı

**Affiliations:** Department of Sculpture, Faculty of Fine Arts, Akdeniz University, Antalya, Turkey; Mindpath College Health, Goleta, California, USA; Mayo Clinic, Rochester, Minnesota, USA; Department of Nephrology, Faculty of Medicine, Dokuz Eylül University, Izmir, Turkey; Private Practice, Consultation-Liaison and Addiction Psychiatry, Minnetonka, Minnesota, USA

**Keywords:** Suicide, sculpture, psychiatry, art history, qualitative thematic analysis

## Abstract

**Background:**

Although clinically framed as a public health concern, the meanings of suicide are fundamentally shaped by cultural narratives and visual representations.

**Aims:**

To examine the evolution of sculptural representations of suicide from antiquity to the present, and to interpret these works through psychiatric and sociological lenses relevant to contemporary clinical and public health discourse.

**Method:**

A structured search across PubMed, Scopus and Web of Science (up to August 2025) was integrated with museum archives and art-historical catalogues. Selected sculptural works were analysed as interpretive case studies using iconographic, semiotic and contextual approaches. Interpretation focused on affective and relational processes such as psychological pain (psychache), shame, entrapment and social disconnection prioritising cultural formulation over retrospective diagnostic attribution in historical cases.

**Results:**

Sculptural representations frame suicide through shifting moral and social logics: from honour-bound self-death in antiquity and virtue-coded narratives in the early-modern period to interiority and estrangement in modernity. Contemporary public installations shift this focus towards visibility, urban space and prevention. These works externalise private suffering and structural conditions (e.g. isolation, stigma), actively shaping collective imaginaries of self-destruction.

**Conclusions:**

Sculpture provides a unique medium for the translation of individual suffering and the collective meanings of suicide into public form. An interdisciplinary reading of these works supports culturally informed clinical reflection and contributes to ethically attentive public communication and prevention strategies.

Suicide is a leading cause of preventable mortality and a central concern of clinical psychiatry, yet it is equally a profound social and cultural phenomenon shaped by collective narratives, moral regimes and public symbols. Durkheim conceptualised suicide as a ‘social fact’ structured by forces of integration and regulation,^1^ and contemporary research continues to demonstrate strong associations between suicidality and social conditions such as isolation, loneliness and exclusion.^2^ Despite this established psychosocial dimension, the visual and plastic arts domains in which suffering, shame, duty and stigma are symbolically negotiated remain comparatively underexamined within clinical, educational and public health discourse. Art possesses a distinctive capacity to reshape perception and articulate experiences for which verbal language proves insufficient,^3^ and artistic representations of suicide can shape public imaginaries by influencing stigma, help-seeking behaviour and ethical reflection; at the same time, inadequately contextualised depictions risk sensationalisation.^4^ Current scholarship does not interpret artworks engaging with suicide as incitements to self-harm, but rather as visual narratives that externalise distress, cultivate awareness and invite affective and cognitive engagement.^3^ Historically, interpretations of suicide in art have varied considerably: Western traditions have often aestheticised the act through heroic or sacrificial figures, whereas lived experiences of suicide have frequently encountered moral condemnation and enduring stigma.^4^ In this sense, sculptural representations function as cultural documents that register shifting historical understandings of agency, coercion and the social determinants of distress. The present study analyses selected sculptural works from antiquity to the contemporary period through integrated psychiatric and sociological frameworks. The aim is not to retrospectively diagnose historical figures, but to translate visual motifs into clinically meaningful and ethically attuned concepts, thereby contributing to cultural formulation, professional training and public communication strategies within the broader field of suicide prevention.

## Method

This study used a qualitative, interdisciplinary design to analyse representations of suicide in sculpture and to explore their interpretation in relation to psychiatric and sociological frameworks.

A structured literature search was conducted in PubMed, Scopus and Web of Science covering publications up to January 2026. Search terms combined suicide-related keywords (e.g. suicide, self-killing, ‘psychache’, shame, entrapment) with art-related terms (e.g. sculpture, representation, visual art) using Boolean operators. Additional sources included reference lists, museum archives and institutional materials.

Inclusion criteria comprised the following: (a) peer-reviewed psychiatric and sociological literature relevant to suicide; (b) documented sculptural works depicting suicide; and (c) archival or institutional materials necessary for contextualisation. Non-sculptural works or insufficiently documented cases were excluded.

Sculptural works were selected purposively rather than exhaustively, focusing on historically documented works that directly depict suicide or its threshold and represent different periods and modes of representation.

Visual materials were analysed as interpretive case studies using iconographic, semiotic and contextual reading, following a consistent sequence: formal description; iconographic and narrative framing; sociohistorical context; and cautious translation into clinically relevant constructs.

Psychiatric interpretation prioritised transdiagnostic and prevention-relevant constructs (e.g. psychological pain, shame, entrapment), whereas diagnostic language was used sparingly and retrospective diagnosis was avoided.

### Ethical standards

This study involved no human participants and used no patient-level data; therefore, ethics committee approval and informed consent were not required.

## Results

### The visualised mental state of suicide in sculpture

Art holds the power to materialise those experiences that lapse into silence at the very limits of language. Within the realm of sculpture, suicide is rendered not simply as an internal crisis, but as a spatial manifestation of cultural codes such as honour, shame or alienation deeply etched into the collective memory. Whereas the classical traditions of Western thought framed this act within a narrative of civic duty or virtuous sacrifice that overshadowed individual agency, contemporary practices pivot, re-reading the subject through the lens of human vulnerability, collective responsibility and the urgent necessity of social repair.

### Antiquity and the classical period

#### The Suicide of Ajax

**Fig. 1:**
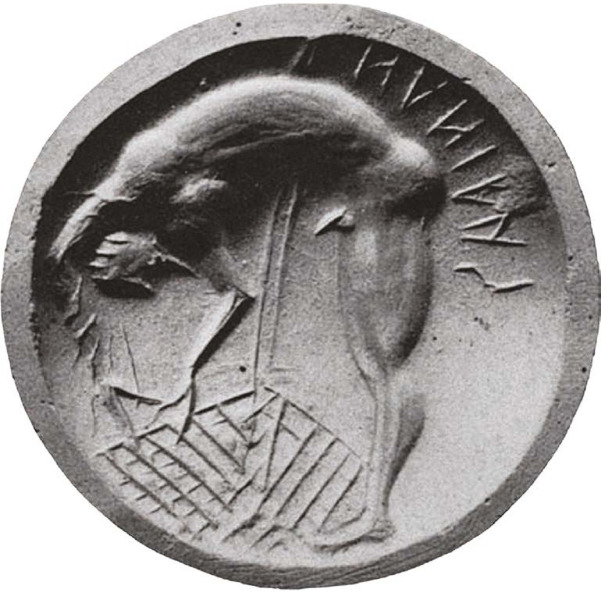
*The Death of Ajax*, seal from Corinth, c. 700 BC. Musée du Louvre, Paris.^6^

The historical representation of suicide is closely associated with Ajax, renowned for his heroic death. This demise, depicted across vases, jewellery and sculptures,^5^ finds its earliest expression in seals showing a gladiatorial stance where the sword symbolises deliberate choice^3^ (Fig. 1). In these sculptural works, Ajax is frequently captured at the threshold of the act: a tensed, forward-leaning body condenses decision and collapse into a single, frozen moment. Here, the sword functions not merely as a weapon but as a material hinge through which agency is staged. In this configuration, honour and psychic unravelling appear inseparable as formal properties of the figure.

Iconographically, the pre-act posture, the deliberate placement of the blade and sustained muscular tension mark a threshold of intent. Crucially, this reading is grounded in formal and compositional cues rather than inferences about subjective motivation. Accordingly, the language of agency, how the act is represented, is analytically distinguished from the narrative explanation of why it occurs. Such interpretive restraint is necessary to avoid the pitfalls of retrospective diagnosis while preserving analytical clarity.

**Fig. 2:**
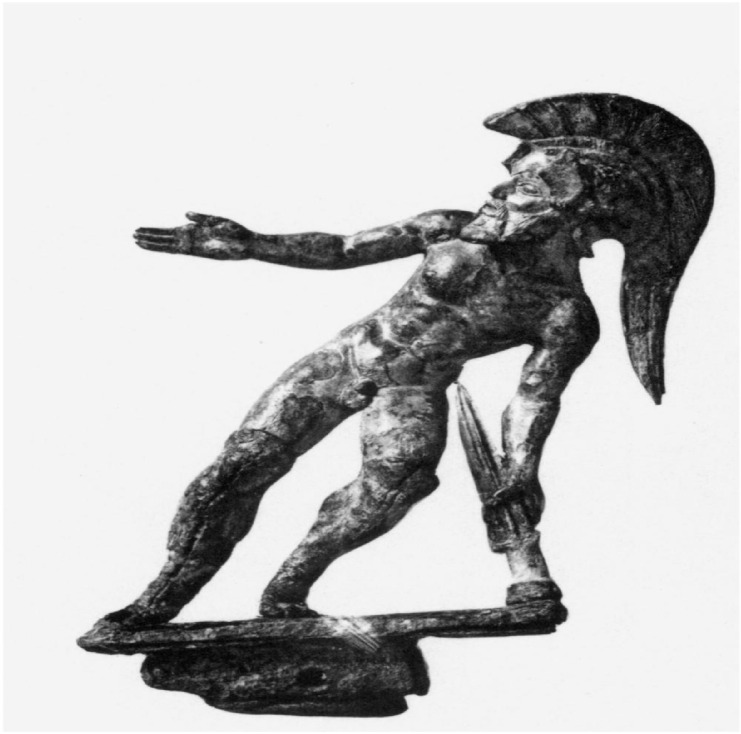
*The Suicide of Ajax*, bronze statue from Populonia, 5th century BC (adapted from ^8^).

**Fig. 3:**
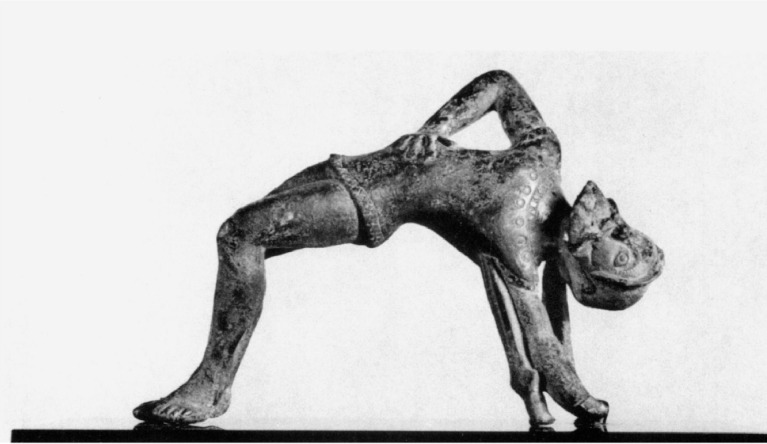
Bronze Etruscan statuette of Ajax from the R. Käppeli Collection. Basel, Antikenmuseum Ka 531. Image: Claire Niggli (adapted from ^8^).

There are two principal narratives regarding Ajax’s suicide. Pindar attributes it to a situation of dishonour. Sophocles, however, offers a more detailed version: after Achilles’ armour is awarded to Odysseus, Ajax becomes enraged, loses his sanity and slaughters sheep, mistaking them for enemies. Upon realising his actions, he takes his own life.^3^ Another interpretation frames his suicide within the context of narcissistic injury and revenge. Lansky argues that Ajax, feeling wronged, withdrew from his social bonds in shame and anger, which precipitated his tragic end.^7^ The bronze statue of Ajax unearthed in Populonia (Fig. 2) captures his despair, depicting him turning his face away at the moment of self-destruction.^3^ In another Etruscan statuette (Fig. 3), the tension within his body conveys the merging of heroic spirit with tragic fate.^8^

Van Hooff characterises Ajax’s suicide as a fundamentally heroic act, a strategic reassertion of agency aimed at reclaiming lost honour.^5^ This historical paradigm finds a compelling parallel in contemporary clinical literature, which identifies a profound link among intensified shame, narcissistic vulnerability and suicidal ideation. Within this framework, shame-related narcissistic injuries are described not merely as emotional states but as structural ruptures capable of accelerating rapid psychic disintegration.^7,9^ By approaching this thematic continuum through Shneidman’s concept of psychache (intolerable psychological pain) rather than imposing restrictive, retrospective diagnostic labels, the subjective logic of the act remains visible without collapsing artistic representation into clinical data. The prioritisation of psychache mechanics enables a more granular modelling of suicidal processes, reaching into conceptual depths frequently overlooked by traditional psychological autopsies, largely restricted to post-mortem medical data.^10^

### The Galatian warriors and the Ludovisi ‘suicidal Gaul’ and his wife

In ancient Greek and Roman contexts, suicide was closely tied to social status. Although voluntary death could be accepted as honourable among elites, it was often condemned among lower classes and enslaved individuals, largely because of its economic implications.^11^ Suicide thus functioned not solely as an individual decision but as a practice shaped by social hierarchy and moral order. In honour cultures, self-death could operate as a means of restoring threatened dignity or social standing, particularly following humiliation.^12^ A historical tension between pagan and early Christian thought framed voluntary death as either heroic and rational^13^ or, in early Christianity, as subordinated to martyrdom and the sanctity of life.^3^

These logics are reflected in Hellenistic victory monuments erected after Attalos I’s defeat of the Galatians around 230 BCE. Pergamene sculpture portrayed defeated enemies with striking dignity while simultaneously constructing narratives of honour, sacrifice and cultural otherness.^14^

**Fig. 4:**
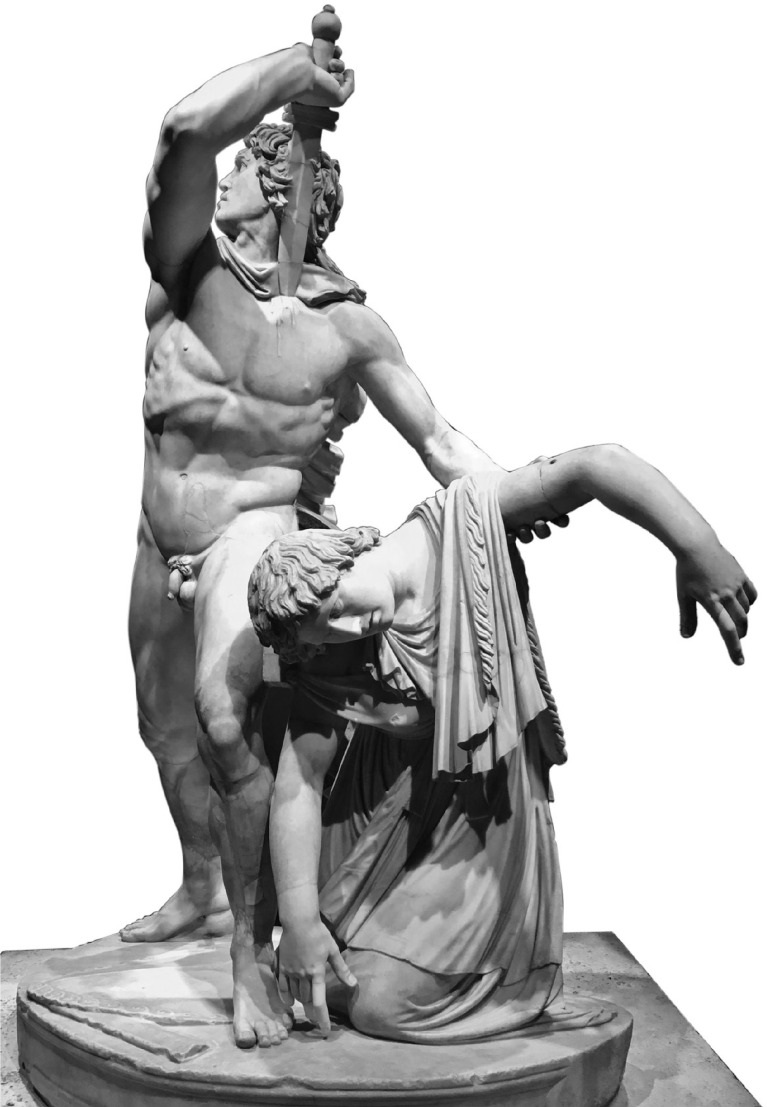
The Ludovisi ‘Suicidal Gaul’ and his wife.^16^

The Roman copy of the Hellenistic statue *Gaul Killing His Wife and Himself* (Fig. 4) exemplifies this framing. The Gallic warrior kills his wife to prevent her capture and sexual violence and then turns the weapon towards himself.^15^

The composition binds two bodies into a single sculptural knot. The male figure’s muscular tension conveys both strength and irreversibility, with the woman’s slackened posture registering the finality of death. Bodily contact produces a tragic economy in which honour, coercion and vulnerability are held within a single gestural configuration. Read in this way, the woman’s posture need not signify ‘surrender’ but can be understood as the aftermath of being killed, where apparent ‘support’ instead indexes coercion and injury. The male figure, poised at the threshold of self-inflicted death, introduces a temporal sequence in which the woman’s death is already inscribed, and suicide remains imminent.

Departing from the idealising balance of Classical Greek sculpture, the work frames voluntary death as an honour-coded alternative to enslavement and sexual violence.^15^ As part of Attalid victory imagery, it functions simultaneously as triumphal display and a ‘merciful’ representation of the defeated enemy.^3,14^

From a contemporary clinical perspective, this configuration resonates with phenomena later categorised as altruistic or joint self-death, although such parallels function here as thematic echoes rather than diagnostic equivalents. In honour cultures, where the failure to uphold masculine ideals could render an individual a ‘social burden’, suicide emerges as an ‘honourable exit’, a final effort to shield familial reputation.^17^ Within the extremity of defeat, these acts reflect a moral logic where self-destruction is framed as a desperate preservation of loved ones from perceived greater harms, demonstrating how deeply cultural scripts dictate the boundaries of the unthinkable.

### The Suicide of Dido

**Fig. 5:**
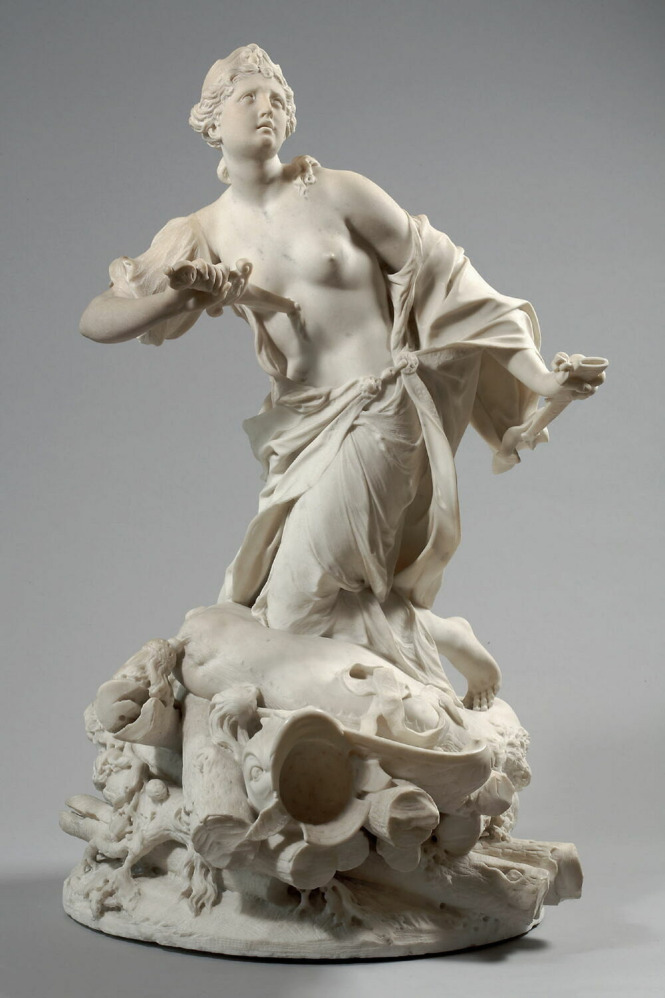
*Death of Dido*, Augustin Cayot, 1711. Louvre Collections. Cayot A. La mort de Didon [statuette]. Paris: Musée du Louvre; 1711. Available from: ark:/53355/cl010091978.

Alongside suicides shaped by romantic despair, mythological narratives of self-death grounded in heroism and sacrifice occupy a prominent place in the plastic arts. *The Suicide of Dido* (Fig. 5) holds a liminal position between these registers. In Roman cultural thought, suicide could be understood as an escape from shame, an expression of profound grief or, when aligned with civic or moral duty, an honourable act.^18^

In the canonical epic tradition, Dido’s death follows a decisive rupture in an intimate bond and is staged through concealment, preparation and a final threshold moment. Rather than rehearsing the full narrative sequence, sculptural interpretation can focus on how this tradition is condensed into form and gesture.

In sculptural representations, such as Cayot’s work (Fig. 5), Dido is captured in a charged pre-act moment. The upward opening of the torso, combined with intense bodily torsion and the approaching blade, suspends the figure between a final appeal and irrevocable resolve. Here, drapery and posture externalise affect as spatial tension, rendering abandonment and inner conflict visible through form rather than mere narrative explanation.

Art-historical scholarship notes that such representations concentrate on the instant preceding the act to heighten dramatic tension and moral ambiguity.^5^ Rather than adjudicating divergent textual versions of Dido’s death, the sculpture condenses uncertainty into bodily hesitation, staging grief as physical strain.

From a contemporary clinical perspective, Dido’s suicide is best approached through the affective processes of abandonment, hopelessness and destabilised attachment rather than retrospective diagnosis. Separation and perceived betrayal are well-established precipitants of suicidal crises, particularly when the weight of loss overwhelms motivational ties to life.^19^ Psychological pain (psychache), perceived inescapability and abandonment-related hopelessness constitute central triggers for suicidal behaviour,^20^ providing a more coherent bridge between the visual language of the sculpture and clinical understanding than discrete psychiatric categories. Whereas mythological frames often elevate such deaths through heroism, modern perspectives emphasise how acute affective collapse following relational rupture may erode the capacity to endure. Rather than ‘explaining’ the act through diagnostic labels, the sculptural representation of Dido renders visible the emotional dynamics of abandonment, inviting reflection on the cultural scripts that shape how suffering is made meaningful.

### Lucretia

*Lucretia*’s sculptural pose translates a rigid moral narrative into bodily rhetoric. Compositional balance, the subtle recoil of the limbs and the axis of the weapon converge to stage a public language of honour under duress. This suspended movement condenses both physical pain and psychological determination, directing attention to the social scripts that frame self-death as the only viable exit from unbearable shame.

**Fig. 6:**
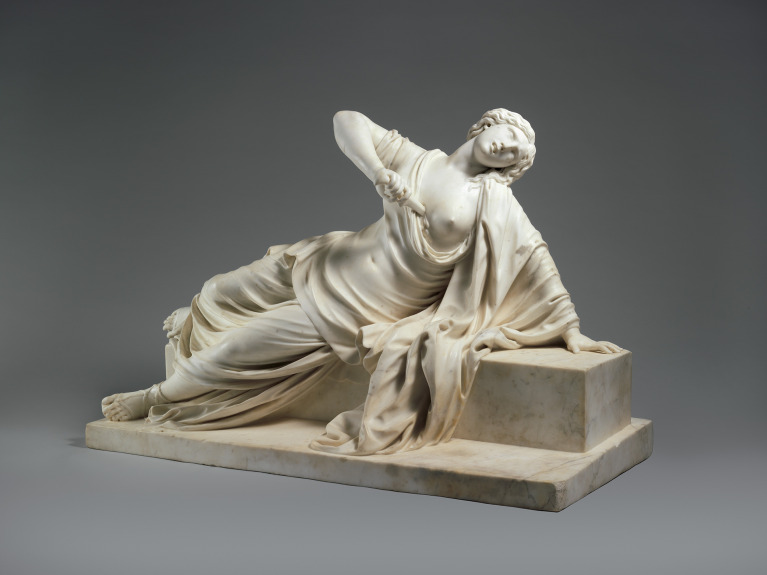
*Lucretia*, Philippe Bertrand, 1704 or earlier. The Met Collection. Bertrand P. Lucretia [marble sculpture]. New York: Metropolitan Museum of Art; 1704. Available from: https://www.metmuseum.org/art/collection/search/212215.

In Renaissance and early-modern visual culture, suicide was often framed as an act of patriotism, moral integrity or sacrifice. Lucretia emerged as a paradigmatic figure of honour-based self-death: after her rape by Sextus Tarquinius, she declared suicide the only means to reclaim her dignity and called for vengeance before taking her life. Her narrative, centred on the irrevocable loss of honour and demand for justice, endured for centuries in artistic tradition, presenting her act as morally exemplary despite her family’s insistence on her innocence.^4^ Whereas this tragic theme recurred frequently in two-dimensional media, it appeared less often in sculpture. In Bertrand’s representation (Fig. 6), Lucretia is not depicted in passive collapse; instead, she occupies a supported, self-sustaining posture. One arm braces her weight against the block while the torso remains partially upright and the head inclines forward. This configuration sustains a deliberate tension between support and descent, staging a moment of conscious resolve rather than surrender.

Where such representations frame the act as rational and virtuous within a moral regime, a contemporary clinical perspective does not presume psychiatric disorder; instead, it foregrounds processes of shame, coercion, fear and social constraint. Read in this way, the sculpture is approached not as evidence of a diagnosable condition but as a visual articulation of how honour-based moral orders can transform sexual violence into self-directed death.^1,21^

### The Death of Cleopatra

**Fig. 7:**
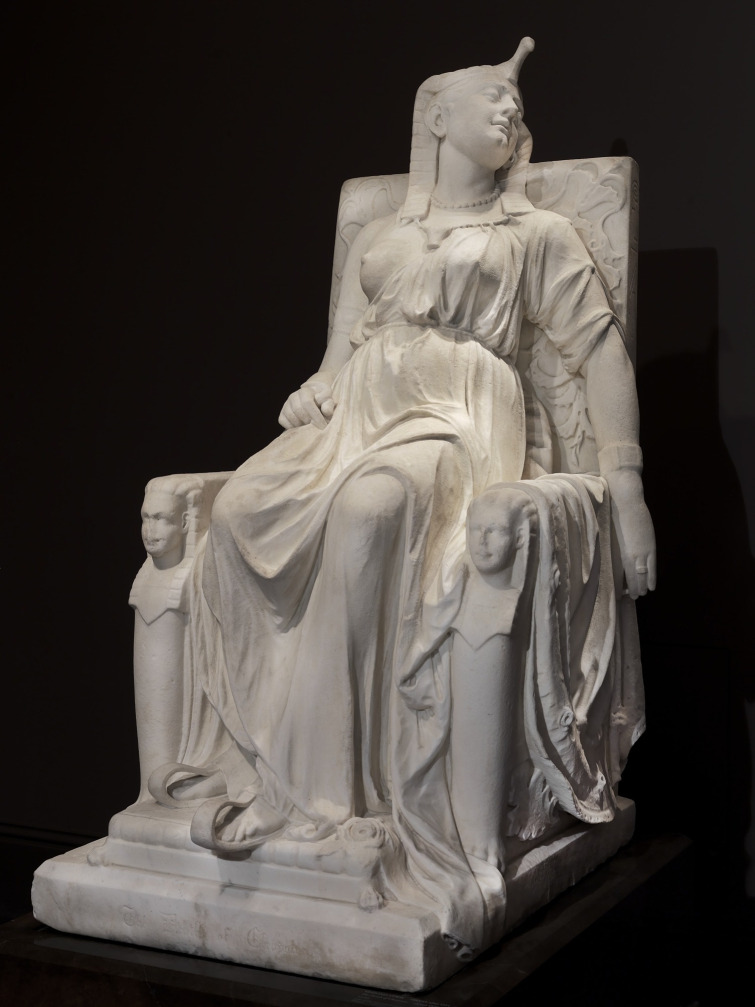
Edmonia Lewis, *The Death of Cleopatra*, 1876. Smithsonian American Art Museum Smithsonian American Art Museum. *The Death of Cleopatra* [marble sculpture]. Washington (DC): Smithsonian American Art Museum; 1876. Available from: https://americanart.si.edu/artwork/death-cleopatra-33878.

In the 19th century, sculptors continued to draw on the enduring myths surrounding Cleopatra’s beauty, power and sovereignty. Edmonia Lewis’s *The Death of Cleopatra* (Fig. 7), executed in a neoclassical idiom, offers a distinct interpretation of this tragic end. Rather than depicting the dramatic act itself, Lewis presents the queen in the heavy stillness of the aftermath, inflecting the work with reflections on power and constrained agency.^22^

Lewis’s Cleopatra is seated upon her throne, which functions not as a neutral support but as a rigid political structure. Her body remains within this apparatus of sovereignty even as life withdraws from it, creating a profound tension between regal authority and bodily inertia. Whereas the asp held in her hand marks the chosen method of death, the sculpture privileges immobility over action. Unlike classical depictions that capture the moment of self-destruction, this work stages the irrevocability of death through collapse and stillness.

The regalia and drapery stabilise the figure within academic neoclassical conventions. Furthermore, the stark whiteness of the marble reflects the aesthetic norms of the period, which universalised classical whiteness, thereby situating the work within the racialised visual culture of 19th-century Europe and America. The larger-than-life scale intensifies this sense of gravity and finality. The snake emerging from the immobile right hand, coupled with the residual tension in the left arm, fixes the figure at a liminal threshold between a sovereign past and a silent present.^23^

From a contemporary clinical perspective, this representation resonates with affective processes rather than discrete psychiatric categories. Concepts such as defeat, loss of power and entrapment, core elements of the integrated motivational–volitional framework, provide a structured lens through which to understand the psychological weight of the scene.^24^ When invoked, sociological typologies function here as tools for meaning-making rather than diagnostic instruments.^1^ By avoiding retrospective labels such as ‘reactive depression’, the analysis focuses on how the sculpture visualises the collision between political collapse and terminal self-determination.

### Il Suicida

**Fig. 8:**
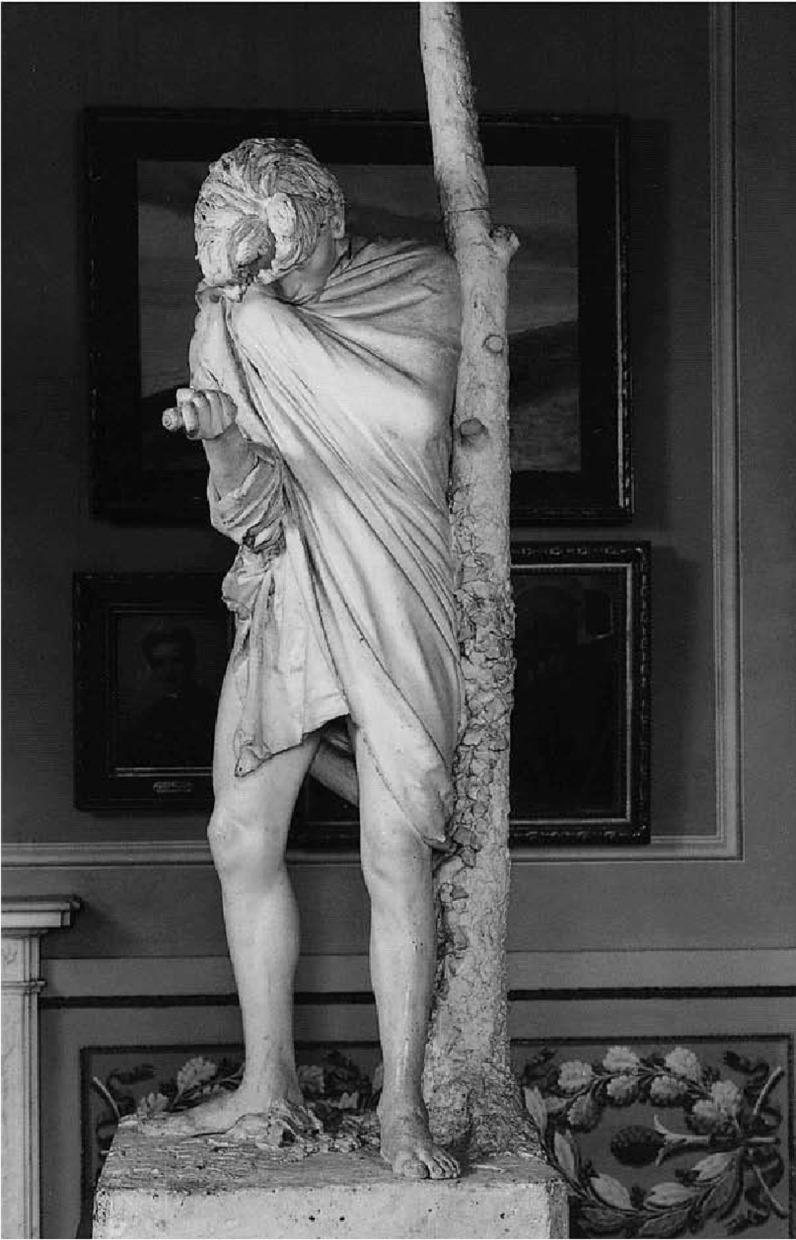
Adriano Cecioni, *Il Suicida*, 1865 (adapted from ^3^).

Adriano Cecioni’s *Il Suicida* (Fig. 8) represents a pivotal moment in 19th-century Italian sculpture, where the focus shifts decisively towards the interior psychological dimensions of self-destruction. The plaster figure is captured in the agonising threshold preceding the act, leaning against a fractured oak trunk. Whereas the oak is a traditional emblem of strength and endurance, its rupture here serves as a potent metaphor: it signifies not merely the loss of vitality, but the inherent brittleness of rigid resilience and endurance that, unable to bend under pressure, inevitably breaks.

Meaning in *Il Suicida* is constructed primarily through bodily rhetoric. The right hand firmly grips the knife while the left draws the garment toward the chin, creating a defensive posture of profound withdrawal. The contraction of the upper body, coupled with visible facial tension, conveys a state of hesitation and inner recoil. This juxtaposition of a physically robust, athletic frame contrasted with a vulnerable, defensive stance reveals an internal dissolution that stands in stark opposition to the heroic resolve seen in classical antiquity.^3^

When read alongside contemporary clinical frameworks, the sculpture resonates with the interpersonal theory of suicide. Joiner’s formulation regarding ‘thwarted belongingness’ finds a visual echo in this figure’s isolation; the work suggests that social pain and perceived disconnection are processed through pathways analogous to physical trauma.^20^ Thus, Cecioni visualises suicide not as a sudden impulse, but as the terminal culmination of a progressive inward collapse.

Sociologically, the work invites a broader interpretation of the individual’s relationship to the collective. The figure’s total detachment from any communal or narrative setting evokes a rupture from the social order, resonating with Durkheim’s theories on weakened social integration and Halbwachs’ emphasis on the social frameworks of meaning.^1,25^ Rather than isolating suicide as a singular event, the sculpture frames it as a multilayered crisis state, a convergence of personal suffering and the erosion of the social bonds that typically anchor the self within the world.^26^

### Awareness-raising representations of suicide in modern and contemporary sculpture

In modern and contemporary contexts, sculptural representations of suicide have undergone a decisive shift, moving away from heroic or moral exemplarity towards themes of visibility, preventability and collective responsibility. Contemporary artists frame suicide not merely as an isolated individual tragedy but as a phenomenon deeply embedded within social organisation, urban space and public health discourse. In this register, sculpture operates as a critical interface between private suffering and public awareness, fundamentally reshaping the dialogue surrounding self-destruction.

Unlike ancient representations, which were inextricably linked to honour codes and collective mythologies, modern works emerge within a paradigm that prioritises intervention and social accountability. The relative absence of preventive discourse in antiquity does not reflect an analytical oversight by the ancients; rather, it indicates a fundamentally different moral and epistemic order. Whereas the ancient sculptor sought to immortalise a virtuous or tragic exit, the contemporary artist often seeks to interrogate the social conditions that precipitate such acts, turning the public monument into a site of reflection on public health and media circulation. By situating these two periods within their respective societal frameworks, the shift from ‘suicide as a heroic script’ to ‘suicide as a preventable crisis’ becomes analytically transparent.

### Suicide Tower

**Fig. 9:**
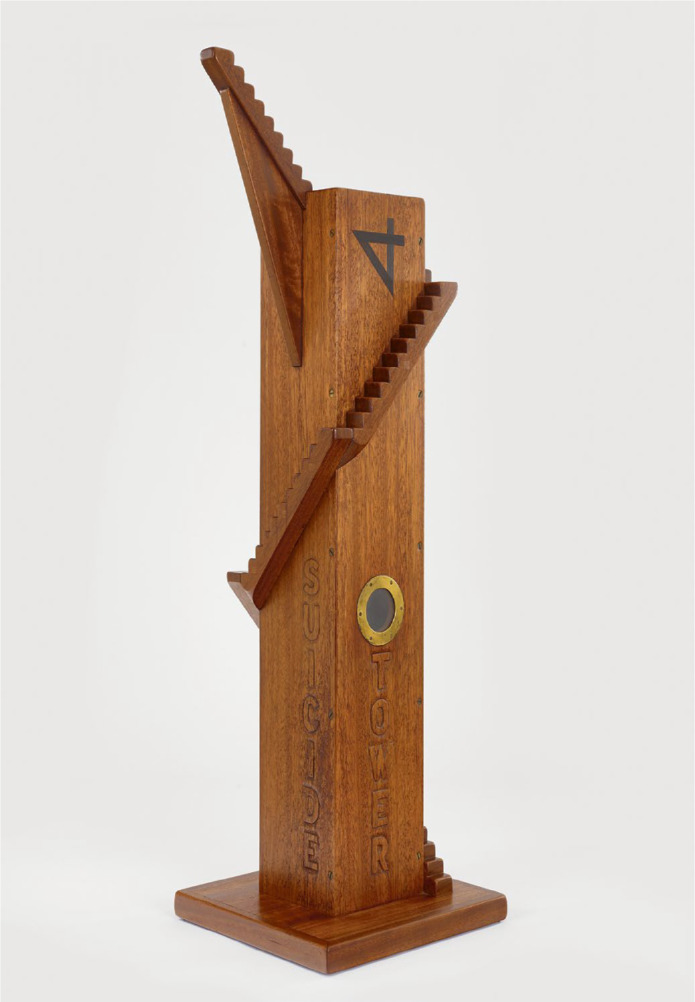
H.C. Westermann, *Suicide Tower*, 1965 (adapted from ^27^).

H.C. Westermann’s *Suicide Tower* (1965) (Fig. 9) eschews the monumentalisation of the act itself, staging instead an arduous ascent towards an uncertain platform. Drawing from the poignant phrase ‘I am going home’, Westermann frames death not as a discrete event but as a suspended possibility, shifting the focus from culmination to process.^27^

Despite the absence of a human figure, the structure evokes a visceral, bodily experience. The steep, exposed climb progressively restricts lateral movement, intensifying a sense of vertical isolation and sustained effort. Although the work may suggest preparation, it equally evokes seclusion, constriction and the sheer strain of upward endurance. In this displacement, suicide is removed from the realm of the dramatic instant and reimagined as a prolonged internal struggle and isolating and difficult progression.

When read alongside contemporary theoretical models that conceptualise suicidal crises as dynamic, time-dependent processes, the sculpture resonates with notions of escalating vulnerability and perceived entrapment without implying diagnostic equivalence. The emphasis on the narrowing physical space visually echoes research into the ‘constriction’ of suicidal states, where perceived options diminish as psychological strain increases.^28^ Ultimately, *Suicide Tower* renders visible an evolving condition of risk rather than a fixed, terminal endpoint.

### Project 84

**Fig. 10:**
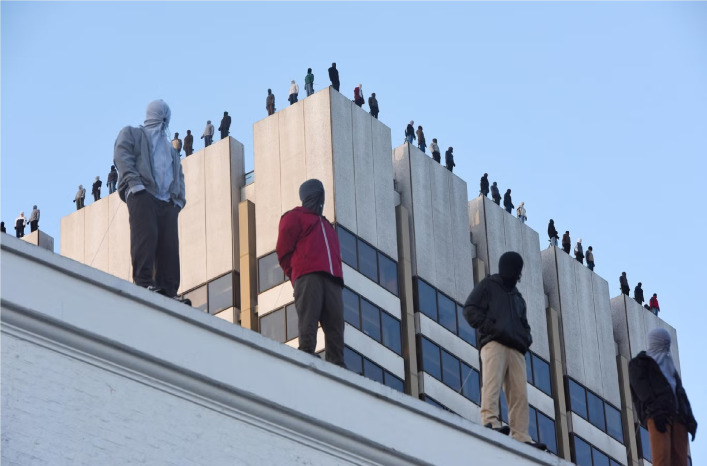
Mark Jenkins, *Project 84*, ITV Building, London, 2018 (adapted from ^29^).

*Project 84* is a public installation comprising 84 life-sized sculptures positioned on the rooftop of the ITV Building in London (Fig. 10). The figure represents the weekly number of men who die by suicide in the UK. Created by Mark Jenkins and collaborators, the work seeks to draw urgent attention to this social crisis through its visual immediacy. By embodying real individuals rather than abstract numbers, the installation transforms statistics into human presence, fostering collective empathy. Supported by the organisation Campaign against Living Miserably, the project frames suicide as a preventable crisis, shifting focus towards social responsibility and improved support for men.^29^

The seriality of the anonymous figures foregrounds scale, whereas the rooftop edge stages vulnerability within the flow of everyday urban circulation. Positioned above the city yet exposed to it, the installation situates the crisis within late-modern architectural space, an environment defined by verticality, visibility and precarity. The edge functions simultaneously as a physical boundary and public spectacle, linking individual vulnerability to the structural tensions of contemporary urban life.

In contrast to ancient representations, which embedded suicide within honour codes and mythic exemplarity, *Project 84* operates within a public health and media ecology that prioritises prevention. Rather than monumentalising self-death, it constructs an ‘awareness aesthetics’ aimed at interrupting social silence. When read alongside clinical research, the rooftop ‘threshold’ resonates with findings that suicidal crises involve acute constriction and fluctuating intensity,^30^ whereas the installation’s emphasis on visibility aligns with the protective role of social connection.^31^ Ultimately, the work functions as a communicative intervention rather than a therapeutic claim, turning the urban horizon into a site of shared accountability.

### Vessel

**Fig. 11:**
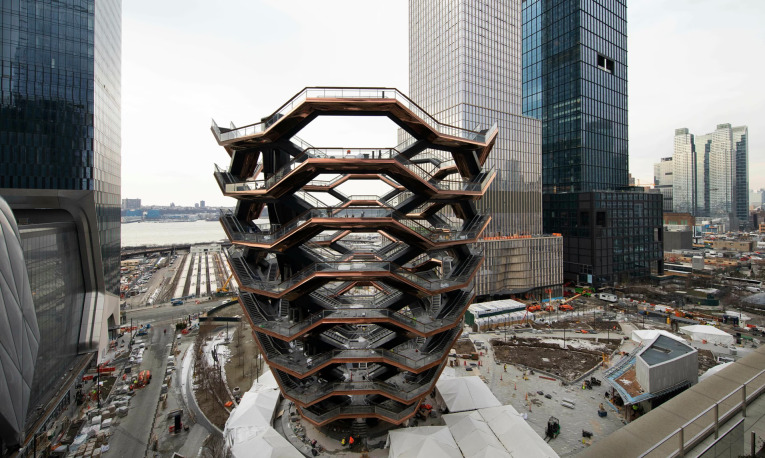
*Vessel*, New York, 2019 (adapted from ^32^).

*Vessel*, designed by Thomas Heatherwick and opened in 2019 at Hudson Yards, New York, was conceived as a monumental, vertically navigable public landmark composed of 154 interlocking staircases (Fig. 11). However, within 29 months of its inauguration, 4 individuals died by suicide after jumping from the structure, leading to its closure and renewed debate about its status as a ‘suicide hotspot’. Although the work contains no figurative reference to self-destruction, its extreme verticality, transparent balustrades and uninterrupted sightlines construct an architecture of exposure in which ascent and vulnerability coexist. Empirical analysis of social media responses to these deaths demonstrates a cumulative intensification of public concern, with later discussions increasingly demanding structural interventions such as higher barriers or netting. In line with broader suicide-prevention literature, restriction of means is identified as one of the most effective strategies at high-risk locations. The case of *Vessel* therefore exposes a tension between architectural spectacle and public health responsibility, revealing how formal openness and aesthetic ambition may require ethical recalibration when recurrent risk becomes structurally evident.^32^

Although *Vessel* does not explicitly represent suicide, its strong verticality, exposed edges and uninterrupted sightlines create a bodily sense of the ‘threshold’, where diminished boundaries and heightened elevation intensify vulnerability. Situated within a dense urban redevelopment context, it operates not as a symbol of death but as an environmental scene in which the aesthetics of ascent meet the precarity of the edge, and the tension between climb and void defines the spatial experience.

Sociologically, this configuration evokes Durkheim’s notion of weakened normative regulation in rapidly shifting urban environments.^1^ From a public health perspective, *Vessel* underscores the fact that accessibility is a modifiable environmental risk factor; evidence from hotspot interventions confirms that physical barriers and means restriction are critical to reducing suicide deaths.^33,34^ Combined with supportive interventions, these structural modifications highlight how modern urban landscapes must balance architectural spectacle with the ethics of public safety.^35^

### Stille Strijd

**Fig. 12:**
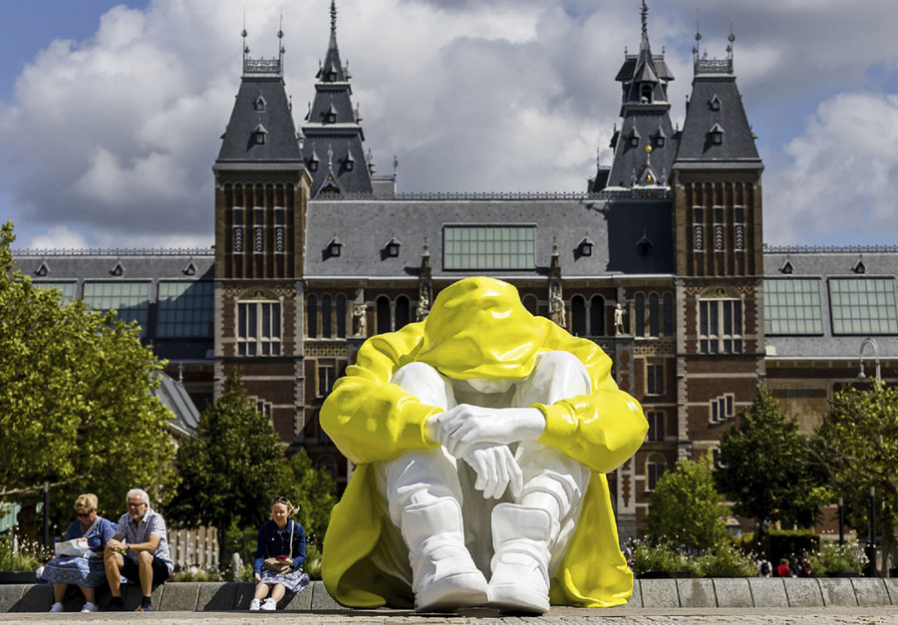
Saskia Stolz, *Stille Strijd*, Utrecht, 2023 (adapted from ^36^).

*Stille Strijd*, created by Saskia Stolz in collaboration with the Dutch suicide prevention foundation Stichting 113 Zelfmoordpreventie, is a monumental public sculpture addressing youth suicide (Fig. 12). First installed in Utrecht in 2023 and subsequently circulated across multiple Dutch cities, the work functions as a mobile national awareness campaign. By placing a seated adolescent figure at an exaggerated scale directly within the flow of everyday urban life, Stolz confronts the public with the embodied, inescapable presence of silent distress.^36^

The sculpture’s aesthetic choices are central to its communicative power. The bright yellow surface maximises visibility in the public square, yet this outward vibrance is contradicted by a collapsed posture, lowered gaze and closed bodily configuration. These formal elements stage a state of profound emotional constriction and psychological withdrawal. The figure is publicly exposed yet internally turned, creating an intense tension between its monumental visibility and represented silence.

Developed in partnership with a prevention foundation, the project explicitly links artistic intervention with public health strategy. Its goal is not memorialisation, but the interruption of stigma and the activation of dialogue. Such public art expands shared attention without prescribing a singular recovery narrative.^37^

This context aligns with research indicating that loneliness and social disconnection, particularly intensified during the COVID-19 pandemic, are core drivers of psychological distress among young people.^38^ Mechanisms such as ‘entrapment’ further illuminate why targeting perceived isolation remains central to prevention.^24^ *Stille Strijd* does not claim direct clinical outcomes; rather, it functions as a symbolic early contact field, making youth distress visible and lowering the threshold for social support.

### Hooded Figure with Teddy Bear

**Fig. 13:**
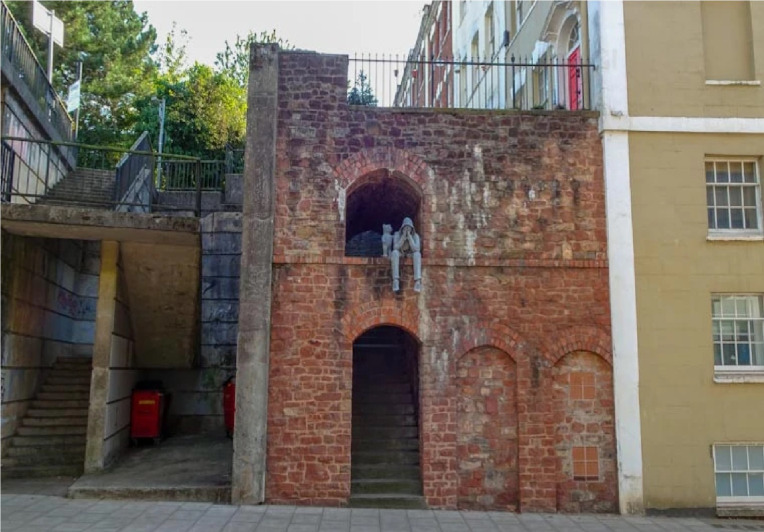
*Hooded Figure with Teddy Bear*, Bristol, 2020 (adapted from ^38^).

In Bristol, a sculpture depicting a hooded figure holding its head in its hands while being comforted by a teddy bear appeared overnight in the city centre, and many interpreted it as marking World Suicide Prevention Day.^39^ The figure’s posture suggests distress, whereas the presence of the teddy bear introduces an element of comfort. The sculpture’s primary force emerges from the tension between bodily closure, the figure’s face hidden in its hands and the proximity of a relational object, staging a conflict between withdrawal and the possibility of contact (Fig. 13).

The surrounding environment is essential to this reading. The unplastered brick wall, worn steps and general sense of neglect situate the figure within an urban space defined by marginality rather than monumentality. Unlike commemorative public works, this sculpture occupies an ordinary, slightly deteriorated setting, reinforcing the invisibility and social sidelining often associated with mental distress. The steps create a subtle threshold dynamic; they suggest potential movement yet anchor the figure in a state of suspension, producing a spatial metaphor for being visible yet socially peripheral.

Read through Winnicott’s concept of the ‘transitional object’, the teddy bear may be understood as mediating between inner crisis and external reality, materialising a minimal ‘holding function’ within an otherwise exposed setting.^40^ At the same time, critical suicide studies caution against reducing such scenes to individual pathology, emphasising how vulnerability is shaped within broader sociospatial contexts.^41^ Ultimately, the sculpture operates as a modest but affectively charged intervention, rendering a ‘silent crisis’ both spatially and socially legible.

### 4 the Love of Go(l)d

**Fig. 14:**
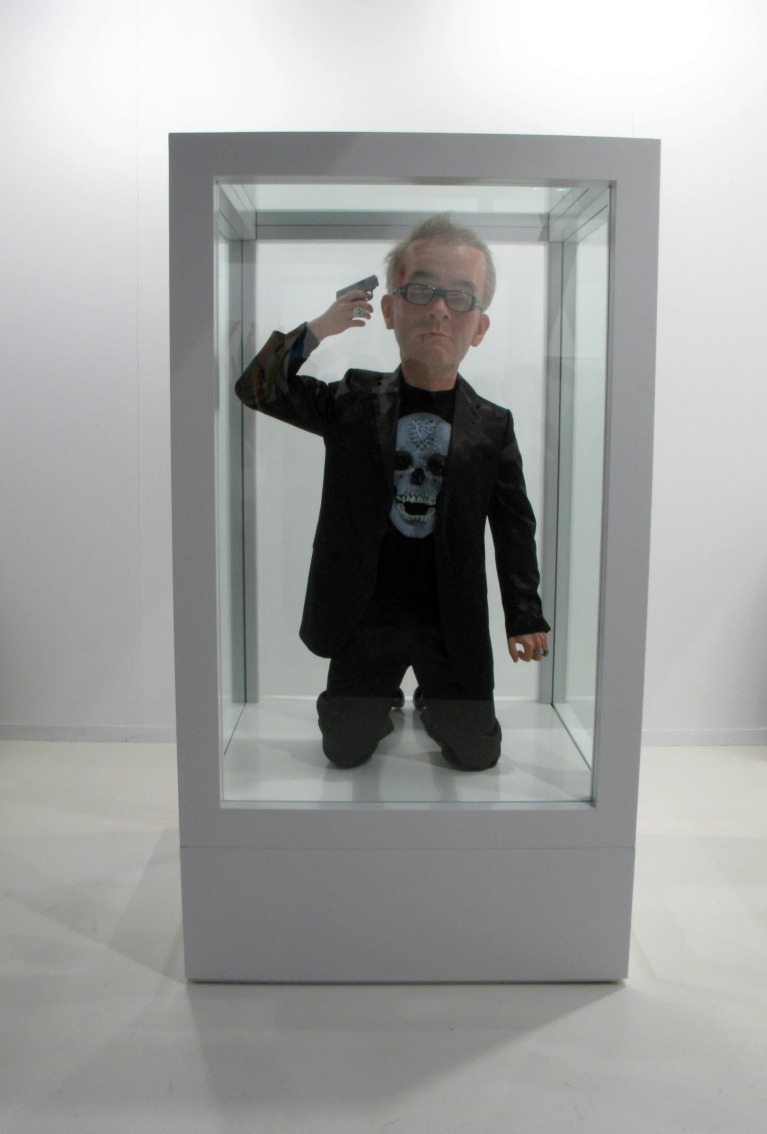
Eugenio Merino, *4 the Love of Go(l)d*, 2009 (adapted from ^42^).

Eugenio Merino’s *4 the Love of Go(l)d* presents a provocative sculptural image of Damien Hirst directing a gun towards his own head (Fig. 14).^42^ Through this gesture, Merino offers an ironic critique of Hirst’s *For the Love of God* (the diamond-encrusted skull) and, more broadly, the market-driven value regimes of contemporary art. The title’s wordplay between ‘gold’ and ‘God’ renders visible a tension between the artist’s physical presence and their market value. Merino’s work suggests a cynical paradox: within a system obsessively oriented towards capital, the artist’s final ‘work’ and their own self-destruction is framed as a potential peak of commodity value, an inversion where the subject’s erasure fuels financial appreciation.^41^

The mode of display is central to this critique. Enclosing the sculpture within a glass vitrine directly references Hirst’s own formaldehyde works, collapsing the ‘display of death’ into a recognisably market-based presentation regime. Here, suicide is recoded not as a romantic or epic endpoint but as an allegory of commodification. In this sense, *4 the Love of Go(l)d* translates the act into the language of the contemporary art economy, where death circulates as a curated image rather than an ethical limit.

This representation resonates with research on the public and media circulation of suicide. Stack’s findings suggest that the high-profile coverage of celebrity suicides can render such acts imitable within certain cultural frameworks.^43^ Read in this way, Merino’s work does not seek a clinical explanation for suicide; instead, it exposes the objectification of the artist’s body within a logic where even death is mobilised as a metaphor for value.

## Discussion

Suicide, although clinically framed as an individual act, manifests in sculpture as a culturally mediated phenomenon shaped by moral regulation, social organisation and historically contingent value systems. Sculpture translates psychological pain, shame and social rupture into visible rhetoric, rendering private distress publicly and spatially legible.

A profound historical trajectory emerges from this analysis. In antiquity and early-modern Europe, self-death was inextricably embedded within honour codes and collective moral regimes, frequently staged as a strategic restoration of dignity or sovereignty. In stark contrast, modern and contemporary works foreground vulnerability, isolation and preventability. This shift is not merely stylistic; it reflects broader transformations associated with industrialisation, urbanisation and the precarious reorganisation of social bonds under late-capitalist conditions.

Recurring spatial motifs edges, ascents, seriality and exposure encode this transition. Whereas ancient works situate suicide within a shared and stable moral order, modern installations such as *Project 84* or *Vessel* stage estrangement within dense yet fragmented urban environments. Here, vulnerability appears not only as personal suffering but as socially situated precarity, where the aesthetics of the ‘threshold’ intersect with weakened social integration.

In this study, Durkheim’s typology functions as a heuristic lens rather than a rigid diagnostic frame. Although his categories resonate with modern contexts marked by diminished regulation, their application to ancient works remains limited by fundamentally different moral cosmologies. This analysis therefore traces shifting ‘moral economies’ rather than imposing anachronistic typological equivalence. Across these diverse periods, sculpture demonstrates that suicide is never purely private: it either affirms collective codes or exposes the fractures within social connection.

By staging thresholds, constrictions and suspended movement, these works translate suicidal risk into an embodied spatial experience, presenting the act as a dynamic process rather than an isolated endpoint. Clinically and educationally, these sculptural representations function as visual case vignettes for cultural formulation. Without relying on categorical diagnosis, they invite a nuanced reflection on how affect, environment and moral narratives intersect. Ultimately, sculptural representation does not merely reflect suicide: it actively participates in shaping the collective imaginary and the social meanings of human endurance and its limits.

## Data Availability

This study did not generate new data. All references and visual materials are derived from publicly available sources, including published articles, books, museum archives and exhibition catalogues.
